# Superior Oxygen Exchange Kinetics on Bi_2_O_3_-Based Mixed Conducting Composites

**DOI:** 10.1021/acsphyschemau.4c00111

**Published:** 2025-02-11

**Authors:** Linn Katinka Emhjellen, Vincent Thoréton, Wen Xing, Reidar Haugsrud

**Affiliations:** † Department of Chemistry, Centre for Materials Science and Nanotechnology, University of Oslo, FERMiO, Gaustadalléen 21, NO-0349 Oslo, Norway; ‡ SINTEF Industry, Sustainable Energy Technology, Pb. 124 Blindern, NO-0314 Oslo, Norway

**Keywords:** oxygen transport membranes, oxygen exchange kinetics, pulse isotope exchange, mixed conducting composites, Bi_2_O_3_-based composites

## Abstract

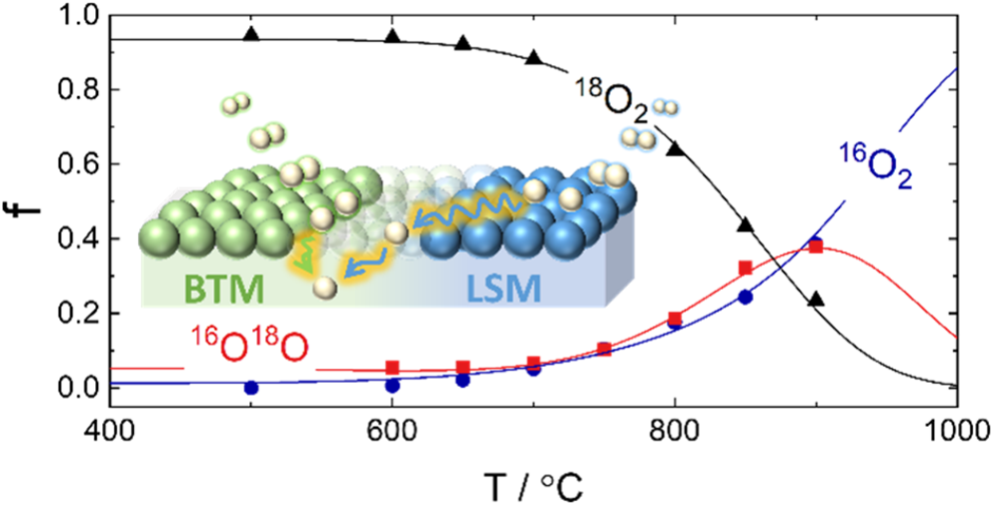

The kinetics of oxygen exchange dictate the rate of redox reactions, which is crucial for electrochemical-based sustainable technologies. In this study, we use isotope exchange pulse responses to elucidate the oxygen exchange mechanism for (Bi_0.8_Tm_0.2_)_2_O_3−δ_ (BTM)–(La_0.8_Sr_0.2_)_0.99_MnO_3−δ_ (LSM) composites. With an optimized composition and microstructure, these composites can achieve polarization resistances below 0.01 Ω·cm^2^ at 700 °C. Analysis of the oxygen exchange rate, 
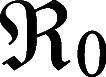
, by splitting it into elementary processes using the serial two-step scheme, demonstrates that both the dissociative adsorption and incorporation of oxygen are accelerated in BTM–LSM compared to the parent phases. Dissociative adsorption of molecular oxygen is rate-limiting below 900 °C in the range 0.002–0.05 atm O_2_ and below 850 °C in 0.21 atm O_2_. Cation interdiffusion or changes in the electronic structure at the interface between the two materials create an electrocatalytically active region spanning 1–40 nm around the BTM–LSM phase boundary. Oxygen exchange coefficients within this region were estimated to be 2–3 orders of magnitude higher compared to those of the entire composite surface. We propose two potential pathways for oxygen exchange in BTM–LSM, with calculated *p*_O_2__ dependencies for each rate-determining step (*rds*). The *p*_O_2__ dependency of 
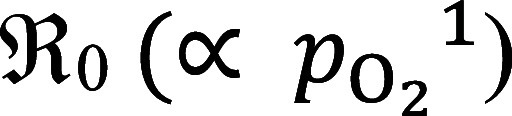
 reveals that molecular oxygen is involved in the *rds*.

## 1 Introduction

Oxygen surface exchange kinetics limits the performance of mixed ionic-electronic conducting (MIECs) oxides in applications essential in technologies for the green shift such as electrodes for sustainable energy conversion technologies and oxygen transport membranes (OTMs). Here, we rationalize the mechanism governing oxygen exchange kinetics of single-phase Bi_2_O_3_-based, (Bi_0.8_Tm_0.2_)_2_O_3−δ_ (BTM), and dual-phase (Bi_0.8_Tm_0.2_)_2_O_3−δ_ (BTM)–(La_0.8_Sr_0.2_)_0.99_MnO_3−δ_ (LSM), which both represent material classes with peak performance in the mentioned technologies.

BTM–LSM composite membranes exhibit oxygen fluxes comparable to those of state-of-the-art single- and dual-phase OTMs.^1−4^ The oxygen flux across membranes as thick as ∼0.1 cm is controlled by mixed surface and bulk kinetics, and limitations from surface kinetics arise gradually with decreasing temperature from 900 to 600 °C. Improving the oxygen exchange kinetics envision further enhanced oxygen permeability, and preliminary data have shown significantly faster surface kinetics for BTM–LSM composites compared to single-phase BTM and LSM.^5^

Surface accumulation of oxygen vacancies and high p-type electronic conductivity account for the relatively fast dissociative adsorption reported for oxygen on LSM surfaces.^6−10^ However, the low oxygen vacancy concentration for this LSM composition with Sr = 0.2 hamper oxygen exchange with bulk,^8,11,12^ and significant oxygen incorporation is not observed until temperatures above 700 °C.^13^ Conversely, bismuth-based oxides show high concentration and transport of oxygen defects, but the oxygen exchange is limited by electronic species.^12,14−20^ Nevertheless, the surface exchange coefficient, *k*, is orders of magnitude higher for Bi_2_O_3_-based materials, which indirectly can be understood through ambipolar oxide ion-electron hole transport, which greatly favors stabilized δ-Bi_2_O_3_.^18,21^

Sluggish oxygen exchange kinetics are challenging in processes involving the electrochemical transport of oxygen species. Consequently, the literature on positrodes for solid oxide electrochemical cells (SOCs) is extensive, where composites with comparable properties to those under study here, e.g., LSM–YSZ, are central. It is worth noting that the surface exchange coefficient of doped Bi_2_O_3_ is 2 to 3 orders of magnitude higher than that of YSZ,^22^ and that Er-substituted Bi_2_O_3_- and Y-substituted Bi_2_O_3_–LSM composites have demonstrated significantly better performance as positrodes in fuel- and electrolyzer cells than conventional YSZ–LSM composites.^23,24^

The enhanced surface exchange kinetics observed for BTM–LSM composites compared to single-phase BTM and LSM reveals a mechanism involving both phases, although the details remain ambiguous.^5^ In this study, oxygen exchange kinetics of single- and dual-phase materials with varying BTM/LSM volume ratios are investigated as a function of temperature and oxygen partial pressure by means of pulse oxygen isotope exchange (PIE). Mechanisms of the oxygen exchange are discussed with basis on the defect structure and surface transport properties of the materials.

## 2 Elementary Reaction Steps in the Oxygen Exchange Mechanism

At thermodynamic equilibrium, the forward and backward reaction rates for the oxygen exchange are equal, with identical *p*_O_2__ dependencies, characterized by the equilibrium oxygen exchange rate, .  is related to *k* by
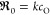
1where *c*_O_ is the volume concentration of oxide ions at equilibrium.  is a lumped parameter representing the overall surface kinetics.^25^ It involves several elementary steps, including the adsorption and dissociation of O_2_ molecules (i.e., dissociative adsorption), the transfer of electrons from the bulk to surface adsorbed oxygen species, and the incorporation of O^2–^ into the oxygen lattice.

 can be determined from ^16^O–^18^O in situ isotope exchange data and broken down to the rates of dissociative adsorption and incorporation according to the two-step scheme.^25−27^ This scheme describes the oxygen exchange reaction as two steps where oxygen is incorporated sequentially (M1) or in parallel (M2). Following the initial charge transfer step (O_2_ ⇌ O_2_^–^ + h^•^), the oxygen molecule may dissociate either with or without the involvement of an oxygen vacancy according to
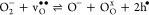
2or
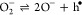
3Here, eq 3 merely reflects the dissociation of oxygen via charge transfer reactions, while eq 2 contains both dissociative adsorption and the incorporation of one oxide ion. The dissociated oxygen ions at the oxide surface may further incorporate into an oxygen vacancy
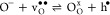
4The combination of eqs 3 and 4 with rate constants  and , respectively, represents a serial two-step scheme (M1) where  is governed by the slowest step. Conversely, eqs 2 and 4 with rate constants  and , respectively, describe a parallel scheme (M2) as oxygen incorporates in both steps. In M2, if  ≫ , most of the oxygen will exchange through eq 2, i.e.,  is dominated by the fastest step, . On the other hand, if  ≫ , the remaining dissociated O^–^ at the oxide surface directly incorporates into an oxygen vacancy and  rate-limits the exchange reaction. So, if the oxygen exchange occurs via M2,  dominates  regardless of the specific situation.

## 3 Experimental Section

(Bi_0.8_Tm_0.2_)_2_O_3−δ_ (BTM) was synthesized by a solid-state reaction of Bi_2_O_3_ and Tm_2_O_3_. Powders of (La_0.8_Sr_0.2_)_0.99_MnO_3−δ_ (LSM) synthesized by spray pyrolysis were purchased from Cerpotech AS. Defined volume ratios of BTM and LSM were mixed in a mortar, pressed into pellets using a 20 mm diameter cylindrical die at 3 tons, and finally sintered at 1000 °C for 10 h.

The surface exchange kinetics of BTM and 60/40, 50/50, 30/70, and 10/90 vol % BTM–LSM were investigated using pulse-response isotope exchange (PIE).^25,26^ Prior to measurements, fine-grained powders of each composition were uniaxially cold pressed (isostatic) to disks and sintered, yielding relative densities of ∼90%. The disks were crushed into coarse powders and sieved to particle sizes of 90–125 μm. The surface area of the powders was determined from Brunauer–Emmett–Teller (BET) measurements.

PIE measurements were conducted in the temperature range 500–900 °C under oxygen partial pressures ranging from 0.002 to 0.21 atm. A ^16^O_2_/N_2_ (0.2–21% ^16^O_2_ in N_2_) gas mixture with a flow rate of 50 mL/min (STP) was used as the carrier gas to which ^18^O_2_/Ar (0.2–21% ^18^O_2_ in Ar) pulses were injected. The pulse response was analyzed at the reactor exit by using a quadrupole mass spectrometer (MS). For more details, see Supporting Information, Section S2.

The phase boundary length in BTM–LSM 50/50 was estimated from SEM micrographs of the composite surface by means of a graphic pixel-based Canny edge detecting algorithm in Python.^28^

## 4 Results

**Figure 1:**
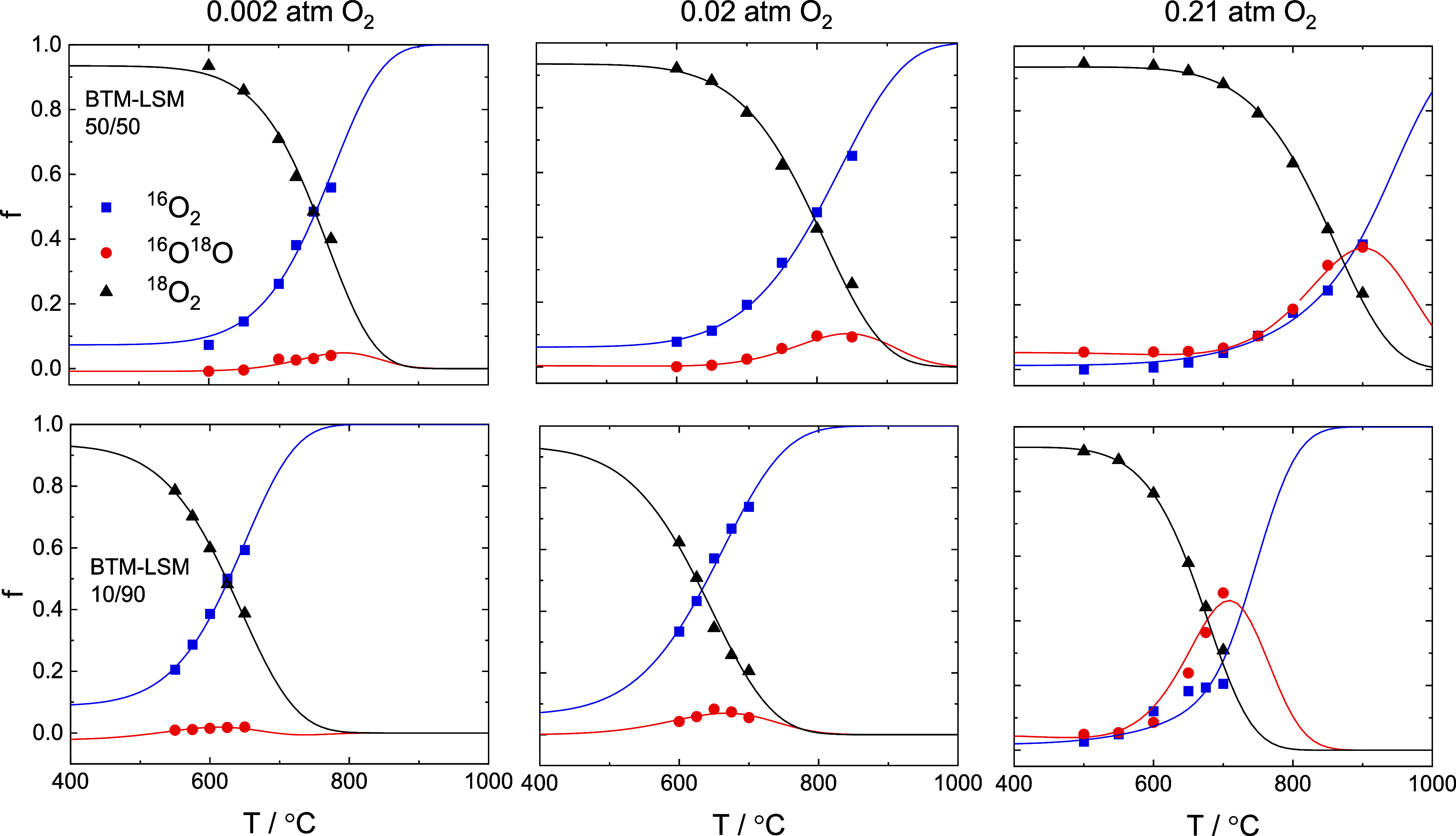
Oxygen isotopologue fractions, *f* (^16^O_2_, ^16^O^18^O, and ^18^O_2_), as a function of temperature from PIE measurements of BTM–LSM 50/50 and 10/90 at 0.002, 0.02, and 0.21 atm O_2_. The lines represent modeled isotope fractions based on a serial two-step scheme for isotopic exchange (M1), assuming constant activation energies for dissociative adsorption and incorporation.^25,26^

Figure 1 shows, by way of example, the fraction of oxygen isotopologues in the pulse response (^16^O_2_, ^16^O^18^O, and ^18^O_2,_ denoted as *f*_32_, *f*_34_, and *f*_36_, respectively) for BTM–LSM 50/50 and 10/90 as a function of temperature in 0.002, 0.02, and 0.21 atm O_2_. The lines represent the modeled isotopologue fractions based on the serial two-step scheme for oxygen exchange (M1),^25,26^ assuming constant activation energies for dissociative adsorption and incorporation rates,  and _,_ respectively. Except for BTM–LSM 10/90 at 0.21 atm O_2_, there is good correspondence between the measured data and the model. The data were also fitted to the parallel two-step scheme (M2) (cf. Supporting Information, Section S1.2). However, more accurate fits were obtained by using M1 as a fitting model.

For BTM–LSM 50/50, the oxygen exchange rate becomes quantifiable from ∼650 °C, as evident from the decrease of *f*_36_. Since there is no gradient in the chemical potential of oxygen in the solid, an equal amount of ^16^O is released into the gas phase by recombination with either ^16^O or ^18^O, increasing *f*_32_ and *f*_34_, accordingly. The ratio of *f*_32_ and *f*_34_ in the pulse response is governed by the relative rates of dissociative adsorption at the sample surface and the incorporation of oxygen into the lattice. For LSM, *f*_34_ was high (>0.5) showing slow incorporation and diffusion of oxygen relative to dissociative adsorption.^7,8^ This behavior leads to unsatisfactory fitting according to the premises of the two-step scheme.^25,26^

**Figure 2:**
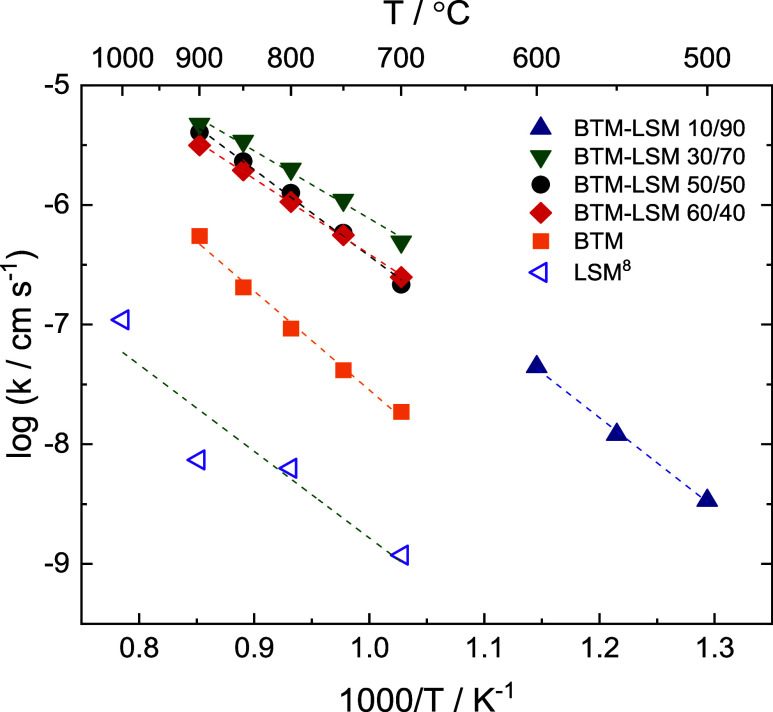
Oxygen exchange coefficient for BTM and BTM–LSM composite membranes with volume ratios of 30/70, 50/50, 60/40,^5^ and 10/90 as a function of inverse temperature in 0.21 atm O_2_. Also shown are data for LSM obtained by ^18^O–^16^O isotope exchange followed by depth profiling in 1 atm O_2_.^8^

Arrhenius plots of *k* for BTM, LSM, and BTM–LSM composites of different BTM/LSM volume ratios in 0.21 atm O_2_ are displayed in Figure 2. Literature data for LSM derived from isotope exchange depth profiling (IEDP) are included for comparison.^8^ Due to the poor fit of the data above 600 °C for the 10/90 composition (cf. Figure 1), its surface exchange coefficient was determined within the temperature range of 500–600 °C, only. BTM and LSM demonstrate an apparent activation energy for oxygen surface exchange of 160 and 130 kJ/mol, respectively,^8^ while corresponding values of the composites fall within the lower range of their constituent phases, varying between 145 and 110 kJ/mol. No clear correlation was observed between BTM/LSM volume ratios and surface exchange rates or the activation energies (cf. Table S1).

**Figure 3:**
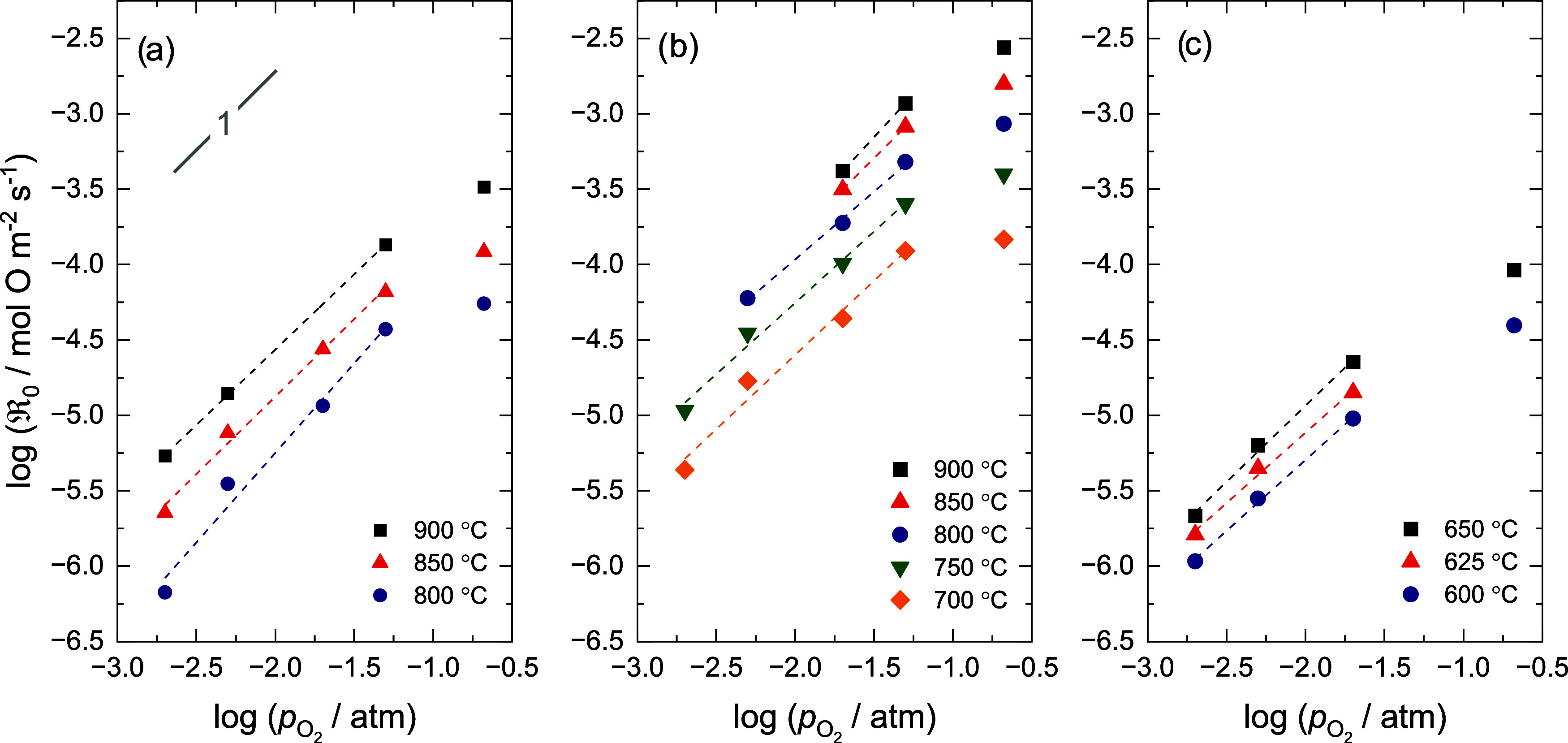
Oxygen exchange rate for (a) BTM, (b) BTM–LSM 50/50, and (c) BTM–LSM 10/90 as a function of *p*_O_2__. Mean slopes with mean standard deviations of 1.07 ± 0.06 for (a), 1.00 ± 0.06 for (b), and 0.96 ± 0.06 for (c), respectively, were obtained from linear regression. Direct proportionality in the log–log representation is indicated as a guide for the eye in the upper left corner of (a). The *x*- and *y*-axes have the same range in all of the graphs.

Figure 3 shows the effect of *p*_O_2__ on  at different temperatures for (a) BTM, (b) BTM–LSM 50/50, and (c) BTM–LSM 10/90.  is essentially proportional to *p*_O_2__ for all materials, except at high oxygen pressures (>0.05 atm O_2_) where the rate constant levels off. The effect is more evident for BTM and the 50/50 composition and increases with decreasing temperature. This could signify a change in the *rds*.

**Figure 4:**
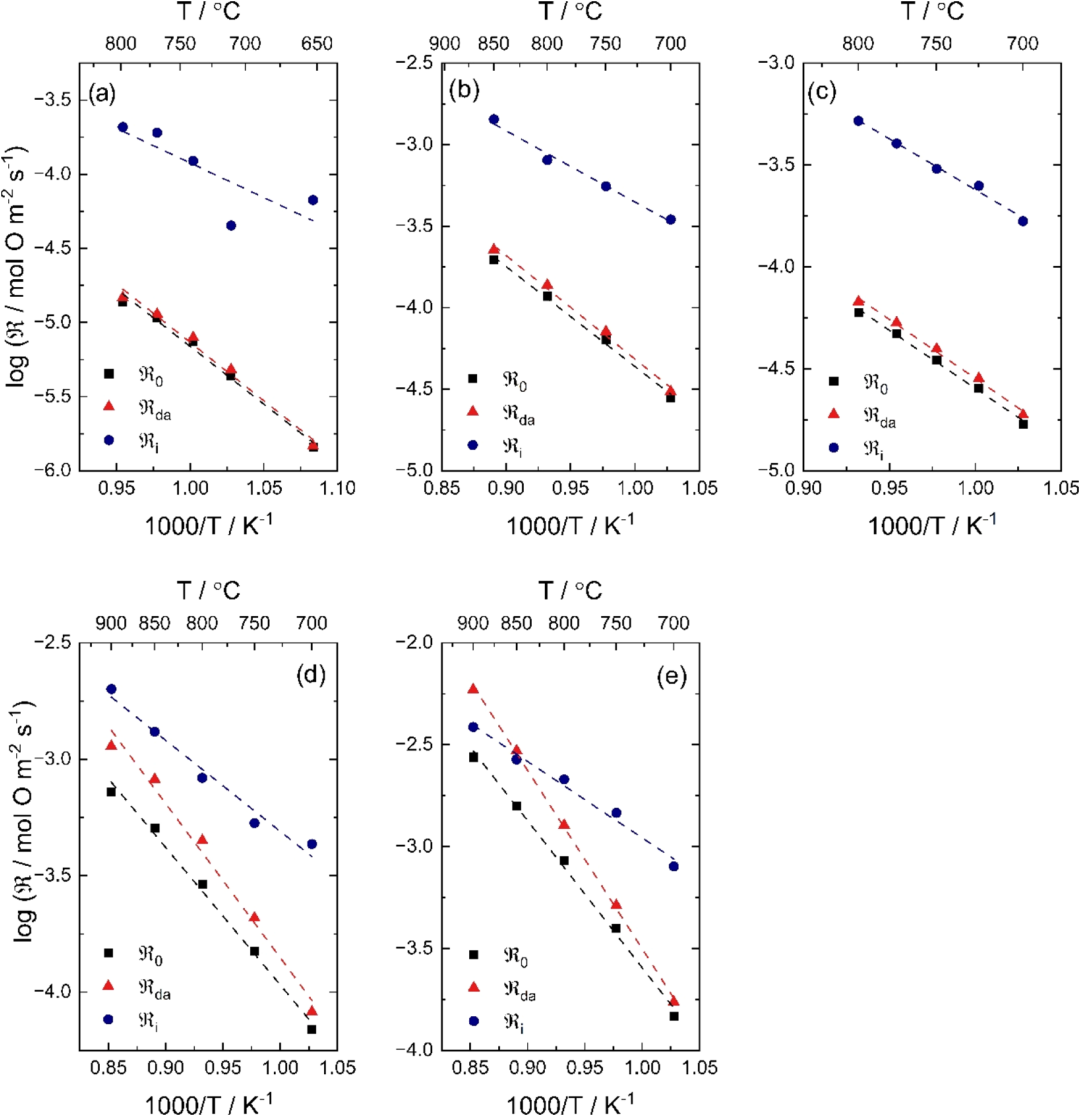
,  and  for BTM–LSM 50/50 as a function of temperature in (a) 0.002, (b) 0.005, (c) 0.02, (d) 0.05, and (e) 0.21 atm O_2_. The data were fitted based on a serial two-step scheme for isotopic exchange (M1).

Figure 4 displays , , and  for BTM–LSM 50/50 as a function of temperature at different oxygen partial pressures. The oxygen exchange is primarily limited by dissociative adsorption except for at ≥850 °C in 0.21 atm O_2_, where the incorporation becomes rate-limiting. Generally, incorporation appears to be less activated compared to dissociative adsorption (cf. Table S2). The difference between  and  is significantly smaller with increasing oxygen partial pressure, reflecting the increased formation of *f*_34_ with increasing *p*_O_2__ in Figure 1. The *p*_O_2__ dependence on  follows that of  within the measured *p*_O_2__ range (cf. Figure S2).  on the other hand, is essentially independent of *p*_O_2__ above 0.02 atm O_2_. The data at lower *p*_O_2__ are disregarded due to errors in the fit of the oxygen isotopologue fractions (cf. Supporting Information, Section S4). Essentially, the same results were obtained upon fitting the data to the parallel two-step scheme (M2);  is primarily constrained by dissociative adsorption, i.e.,  (cf. Figure S1).

Similar behavior was observed for the other compositions, although the change in the rate-limiting process at 0.21 atm O_2_ occurs at different temperatures, i.e., in the range 650–900 °C (not shown). For BTM, the surface exchange rate is also limited by dissociative adsorption below 0.05 atm O_2_. At 0.21 atm O_2_, incorporation becomes rate-limiting in the whole temperature range (cf. Figure S3).

## 5 Discussion

The oxygen exchange rates of BTM–LSM composites are significantly higher compared with those for single-phase BTM and LSM, showing that the two phases jointly promote the surface kinetics. From the *p*_O_2__ dependencies on  and since incorporation is faster than dissociative adsorption, we infer that molecular oxygen species are involved in the *rds* for *p*_O_2__ ≤ 0.05 atm.

**Figure 5:**
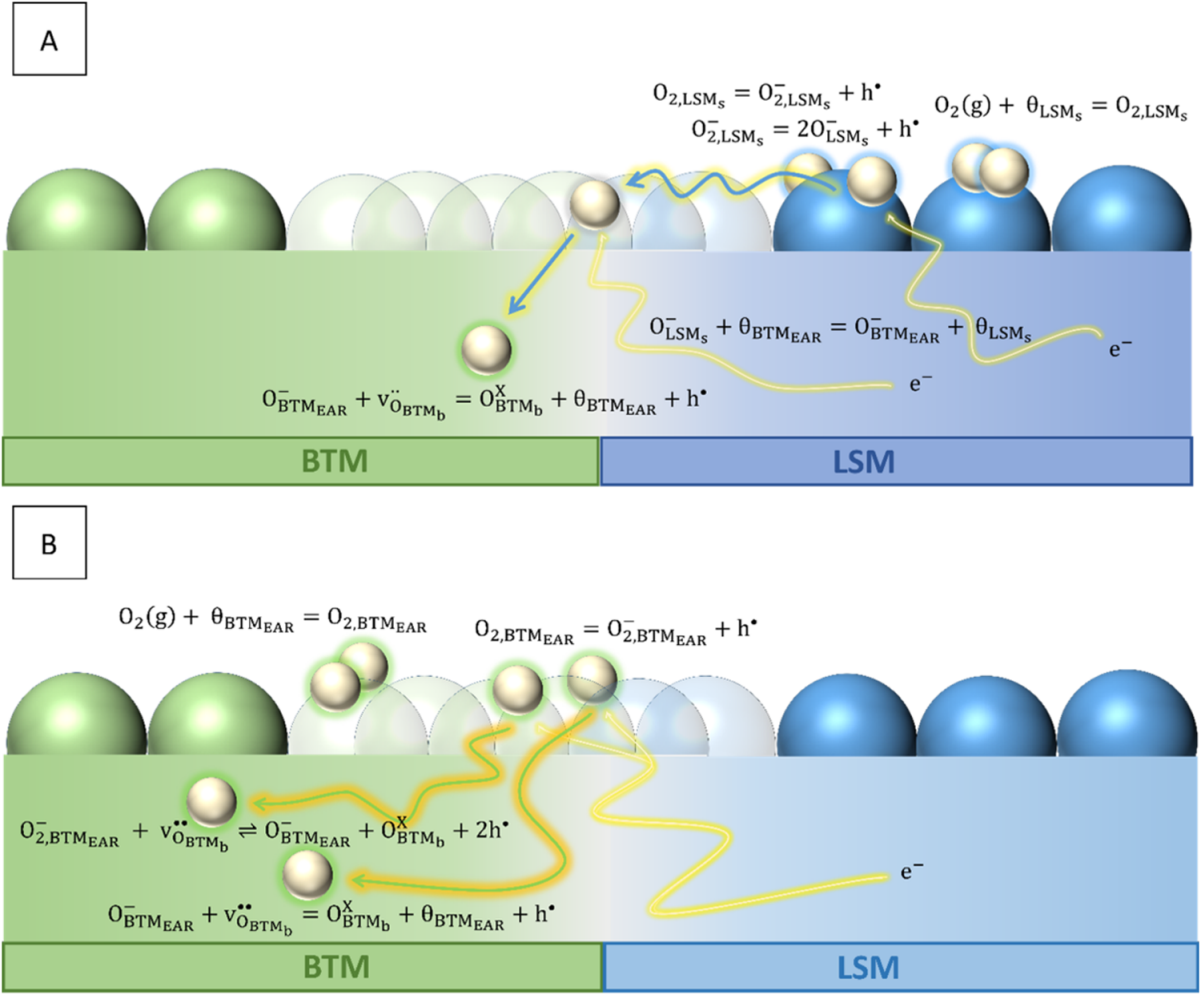
Schematic illustration of two possible pathways for the oxygen exchange mechanism in BTM–LSM (marked A, B). Oxygen gas adsorbs at the surface, *S*, or at the electrocatalytically active region, EAR, at adsorption sites θ_BTM_ and θ_LSM_. An electron transfers to the adsorbates, O_2,BTM_ and O_2,LSM_, prior to dissociation, leaving the species O_2,BTM_^–^ and O_2,LSM_^–^. A second electron transfer occurs during dissociation, yielding O_BTM_^–^ and O_LSM_^–^. Bulk oxygen vacancies are only considered for the BTM phase, v_O_BTM,b__^••^. Effectively neutral oxide ions residing in the oxygen sublattice of BTM are denoted as O_BTM_b__^x^.

Although the functional dependence on  reflects virtually the behavior of dissociative adsorption (cf. Figures 3–5), examining the characteristics of  and  may offer more detailed mechanistic insight. Both dissociative adsorption and incorporation of oxygen are faster in the composite compared to BTM (see the Supporting Information, Section S5). The enhancement of  exceeds that of  (cf. Figure S4). Moreover, the *p*_O_2__ dependence of  is generally lower than that of , approaching invariance at the highest temperatures and oxygen pressures (cf. Figure S2). Incorporation is less activated than dissociative adsorption (Table S1). Due to their different functional dependences on the reaction conditions,  surpasses  at the highest temperatures and oxygen pressures, leading to a gradual shift toward incorporation-limited oxygen exchange (cf. Figure 4e, 0.21 atm O_2_).

A key difference between single-phase materials and composites is the presence of triple-phase boundaries (TPBs) at the interface between the two solids and the gas phase. The interaction between defects in the LSM and BTM creates an electrocatalytically active region (EAR) near the phase boundary that promotes the oxygen exchange reaction. Based on previous literature on dual-phase systems^29−31^ and heterointerfaces,^32−34^ three mechanisms have been suggested that potentially promote the oxygen exchange reaction in BTM–LSM: (i) *spillover* of the species involved in the oxygen exchange, (ii) *cation interdiffusion* between the two phases forming regions near the interface with dissolved foreign cations or the presence of secondary phases, and (iii) *changes in the electronic structure* causing defect segregation and space-charge layers at the interface between BTM and LSM.

A spillover-type mechanism has been suggested to account for the enhanced surface kinetics in dual-phase materials comprising an electronic conductor and an ionic conductor. Here, oxygen species dissociate on the surface of the electronic conductor and migrate across the triple-phase boundaries (TPBs) to the ionic conductor before being incorporated.^30,31,35,36^ This mechanism, known as the “surface path”, is often considered the main route for the oxygen exchange reaction in LSM–YSZ electrodes at open circuit voltage (OCV).^35−38^ If the dissociative adsorption of oxygen on LSM is fast compared to that on BTM, spillover of adsorbed oxygen species, typically O^–^, from LSM to BTM could be relevant. It should be noted that the calculated barrier for O^–^ migration on the surface of LSM is found to be high (∼2 eV).^39,40^

An alternative route is that oxygen species dissociate, incorporate into LSM, and migrate via oxygen vacancies across the phase boundary to BTM, where oxygen diffusion is faster. This is referred to as the “bulk path” in electrode kinetics.^36^ In YSZ–LSM electrodes, this pathway becomes significant under conditions that increase the oxygen vacancy concentration in LSM, such as at high overpotentials or in thin-film samples.^36^ The concentration and mobility of oxygen vacancies in LSM tend to be higher in the surface layers than in the bulk.^9,39^ Hence, oxygen migration via vacancies from LSM to BTM should occur faster in the surface layers than in the bulk and could be viewed as a spillover-type mechanism similar to what is observed in LSCF–ScCeSZ composites.^30^

A previous study on BTM–LSM pellets sintered at 1000 °C for 10 h has demonstrated that there is an approximately 40 nm wide region surrounding the BTM–LSM phase boundary where cation interdiffusion occurs.^4^ On these bases, it is intriguing to speculate whether cation interdiffusion creates an electrocatalytically active region (EAR) near the BTM–LSM interface with enhanced surface kinetics. For instance, valence changes in Mn could increase the number of available electrons for the charge transfer reactions, promoting both dissociative adsorption and the incorporation of oxygen.

Another aspect of cation interdiffusion is the so-called “cleaning effect”.^30,31,41^ Impurities tend to segregate to the surface of fluorite-structured ionic conductors, forming a passivating layer that decelerates the surface kinetics. In dual-phase systems, these impurities may dissolve into the perovskite lattice of the electronic conductor, thereby leaving a clean(er) fluorite surface. This approach is interesting for future research and would benefit from the use of advanced surface analysis techniques.

Surface kinetics relates to the electronic band structure of the oxide surface.^42,43^ For example, the oxide ion affinity, influenced by the position of the Fermi level of the oxide with respect to the adsorbed molecular oxygen, correlates with the rate of dissociative adsorption of oxygen.^42,43^ Hence, changes in the electronic structure at the interface between two materials in a composite lead to distinct surface kinetics compared with the individual phases.

The electrochemical potential difference of charged species in two adjoining materials can lead to defect segregation and the formation of a space charge region characterized by an increased concentration of mobile point defects near the interface.^32,33,42,44,45^ The direction and extent of the interfacial defect segregation depend on the dominating defect disorder of the two oxides and their chemical potentials and electronic structure.^33,46^ When a p-type ionic conductor is combined with a p-type electronic conductor, such as in BTM–LSM, potential electron migration from LSM to BTM upon band alignment at the interface may be charge-compensated by the countermigration of oxygen vacancies. This would lead to the formation of an EAR with elevated concentrations of electrons and oxygen vacancies through heterogeneous doping.^44^ Oxygen vacancy accumulation and Mn reduction have been observed at LSM–YSZ interfaces.^47,48^

Despite the rather different lattice parameters of LSM (3.9 Å^46^) and BTM (5.5 Å^47^), the phase boundary between the two phases is coherent.^4^ The strain induced by the differing unit cell dimensions is presumably accommodated by segregation of oxygen vacancies at the interphase, thereby contributing to the formation of space-charge layers.^33,42,48−50^ A localized increase in the oxygen vacancy concentration could enhance the oxygen exchange kinetics in the EAR by increasing the number of the active defects.

Both (ii) and (iii) involve mechanisms that potentially expand the electrocatalytically active region (EAR) beyond the geometrical three-phase boundary (TPB). Oxide–oxide interfaces with a high concentration of mobile defects build up narrow space charge regions, and significant defect concentration changes are usually within a few nanometers from the phase boundary.^33,45,47,49^ For processes related to cation interdiffusion, one can consider the approximately 40 nm wide region surrounding the BTM–LSM phase boundary as an upper limit.^4^

It has been demonstrated that the extent of the EAR into the subsurface of an oxide with regards to oxygen diffusion depends on the ratio between the oxygen exchange and diffusion coefficient.^50−53^ When this ratio decreases, the contribution of the subsurface layers becomes more important as larger areas around the actual solid–solid boundary are activated. For LSM, the surface kinetics is considerably faster than diffusion (*k*/*D* > 10^4^).^8,54^ Thereby, the surface path predominates and the active region extended into the subsurface layers on the LSM side would be minute relative to the BTM side, even with an increased oxygen vacancy concentration near the phase boundary.

Given that the expansion of the EAR beyond the TPB in BTM–LSM is modest, the exchange kinetics within this region must be remarkably fast to retain the measured surface exchange rates. The overall oxygen exchange coefficient can be represented as the sum of the oxygen exchange coefficients of the individual phases and the EAR in relation to their relative surface areas.^55^ If we, for the sake of illustration, assume that the maximum width of the active area at the surface corresponds to the region with compositional variations (<40 nm), the estimated oxygen exchange coefficient is 2–3 orders of magnitude higher than if the entire composite surface was active (see Supporting Information, Section 6 and Figure S6). The calculated oxygen exchange coefficient for BTM–LSM with an EAR of 1 nm is comparable to that of nanostructured pulsed layer deposition (PLD) thin film LSM with 1 nm wide grain boundaries^54^ but still remains well below the fundamental, upper limit of *k*, determined by the flux of oxygen molecules in the gas phase.^42^

In this context,  should scale with the TPB length. However, although the 10/90 BTM–LSM composition does not form a percolating TPB network, the increase in  observed relative to single-phase BTM is virtually the same as for the 50/50 and 60/40 compositions. The interpretation of this behavior is not straightforward. The significant effects on oxygen exchange for relatively low volume concentrations of a second phase (10–20 vol %) are consistent with literature for other composites.^56−58^ Interestingly, the effective enhancement due to the oxygen exchange rate by introduction of a second phase is observed to be 1–2 orders of magnitude, seemingly independent of the material systems.^31,41,56^ In general, literature data on the correlation between *k* and TPB length are inconclusive,^57−61^ as both parameters depend on the complexity of the microstructure; porosity, particle size, distribution of the constituent phases, and potential impurity phases.

Going into more detail on the oxygen exchange mechanism, two possible reaction pathways for the oxygen exchange are considered for BTM–LSM depending on which phase dominates the dissociative adsorption of oxygen. The possible pathways are sought, schematically illustrated in Figure 5. The successive reaction steps in each pathway are listed in the Supporting Information, Section S7.

First, oxygen exchange can take place via the spillover route (pathway A in Figure 5)—dissociative adsorption on LSM followed by surface migration of O^–^ to BTM where incorporation occurs (cf. Supporting Information, Section S7). We only consider the “surface path” when breaking the reaction down to elementary steps since this is generally regarded as the predominant mechanism for YSZ-LSM electrodes.^35−38^ Also, our estimations show that an increased oxygen vacancy concentration in the LSM yields a minimal extension of the active region into the subsurface layers on the LSM side of the phase boundary. Regardless, the rate-limiting reaction, i.e., dissociative adsorption, occurs via the same elementary steps for both the “surface path” and the “bulk path” (cf. Supporting Information, Section S7).

Experimental and computational studies have shown that the dissociative adsorption on the surface of LSM most likely occurs without the involvement of oxygen vacancies,^39^ even when combined with oxygen ion conductors such as in YSZ–LSM composites.^38,62^ Hence, this reaction pathway is a serial mechanism (M1).

Second, the entire oxygen exchange reaction can occur in the electrocatalytically active region (EAR), for instance, on the BTM side of the EAR where the concentrations of electrons and oxygen vacancies are high (Pathway B in Figure 5, cf. Supporting Information, Section S7). For cubic fluorite structures and perovskites with a high amount of oxygen vacancies, DFT calculations show that the dissociation of molecular oxygen species occurs via oxygen vacancies at the surface.^63−65^ This implies that one oxygen species immediately incorporate upon adsorption, leaving the second one as an adsorbed O^–^ ion, which will either incorporate or recombine with an oxygen species at the surface. Hence, this oxygen exchange reaction is most accurately described by a parallel two-step scheme (M2).

The functional dependences on  of *p*_O_2__ and temperature can provide further insight into the rate-determining step (*rds*) for the oxygen exchange. The *p*_O_2__ dependences for different potentially rate-determining elementary reactions are derived and compared to the experimental observations (cf. Supporting Information, Section S8). For the spillover route (pathway A), both the pure charge transfer step (A1) and the step involving charge transfer and dissociation (A2) in the dissociative adsorption reaction yield  (cf. Supporting Information, Section S8). Given that A2 includes both charge transfer and the breakage of O–O bonds, it is likely the *rds*.^65^ In the case of oxygen exchange within the EAR (pathway B), only the pure charge transfer step in the dissociative adsorption reaction yield  identifying it as the rate-determining (step B1 in Supporting Information, Section S8).

**Figure 6:**
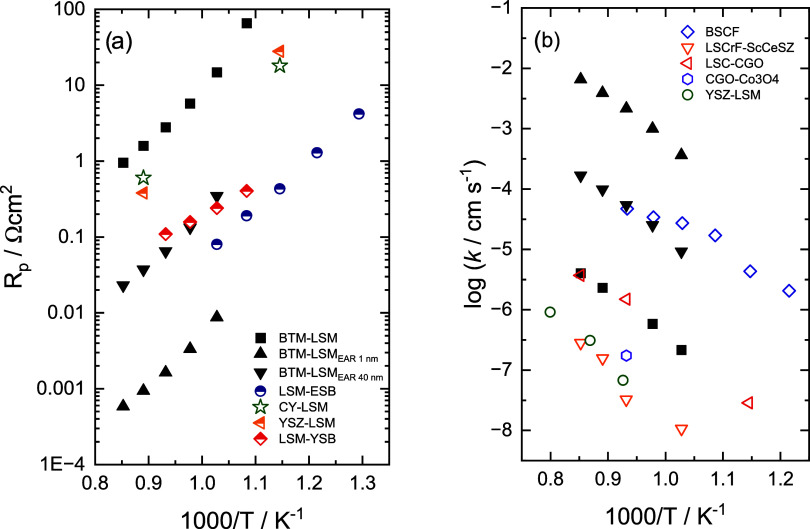
Oxygen exchange coefficients or converted polarization resistances for BTM–LSM 50–50 and the EAR (of 1 and 40 nm width) compared to (a) polarization resistances for Bi_2_O_3_-containing and LSM-based cathode materials^24,67,70^ and (b) tracer oxygen exchange coefficients (k*) for Ba_0.5_Sr_0.5_Co_0.8_Fe_0.2_O_3−δ_ (BSCF)^71^ and several dual-phase OTMs^56,72−74^ as a function of temperature. Note that the values are not directly comparable due to the different measurement techniques and approximations used for obtaining the different data sets.

Maier^66^ derived a relationship between the effective surface exchange coefficient and the polarization resistance of positrodes, which we have utilized to compare the obtained oxygen exchange data to SOFC cathodes used in solid oxide electrochemical energy conversion cells and oxygen gas separation membranes (see Supporting Information, Section S9). Several reports have revealed the high performance of composite cathodes containing Bi_2_O_3_-based oxide ion conductors as major components. Overpotentials are important figures of merit in SOFCs in general, and Figure 6a compares the temperature dependence of polarization resistances for Bi_2_O_3_-containing and LSM-based cathode materials to the values estimated from the oxygen exchange coefficients of BTM–LSM 50–50 and the EAR (of 1 and 40 nm widths). It is evident that with optimized composition and microstructure, BTM–LSM composites have the potential to reach polarization resistances below 0.01 Ωcm^2^, which represents values below those of other material systems.

Lee et al.^67^ investigated the polarization resistance of Er-stabilized Bi_2_O_3_ mixed with 50 wt % LSM through impedance spectroscopy, revealing two contributions to the polarization resistance for ESB–LSM. First, a resistance at high frequency (*R*_H_) associated with incorporation of oxygen in ESB corresponds to charge transfer at the ESB/LSM interface. Second, a resistance at a lower frequency (*R*_L_) reflecting dissociative adsorption and/or surface diffusion on LSM, which was identified as the *rds*. These findings align with the interpretation of the PIE data. The average apparent activation energy of  for BTM–LSM, approximately 1.3 eV, falls within the range of activation energies determined for polarization resistances measured at OCV in symmetric cells with ESB–LSM electrodes (50:50 weight ratio) on an ESB electrolyte (1.18–1.39 eV).^67−69^ The activation energies encountered here for dissociative adsorption and incorporation are higher than the values derived for the processes representing R_L_ and *R*_H_, respectively. It is important to note that differences in the microstructure and experimental conditions, which are inevitably present, can affect these apparent values.

Figure 6b provides a comparison of the oxygen exchange coefficients for the state-of-the-art single-phase OTM Ba_0.5_Sr_0.5_Co_0.8_Fe_0.2_O_3−δ_ (BSCF) with several composite materials. The oxygen exchange rate of EAR is superior to that of the other materials. Considering that the BTM–LSM composites also exhibit ambipolar oxide ion and electron conductivity at state-of-the-art levels, a membrane combining an optimized nanostructured surface morphology with a well-dispersed microstructure in the bulk could yield exceptionally high oxygen fluxes.

## 6 Conclusions

The oxygen exchange rates, , of BTM–LSM composites are significantly higher compared to single-phase BTM and LSM. Both the rates of dissociative adsorption, , and incorporation, , of oxygen are enhanced. The oxygen exchange rate is limited by dissociative adsorption below 900 °C in the oxygen partial pressure range 0.002–0.05 atm O_2_ and below 850 °C in 0.21 atm O_2_. The *p*_O_2__ dependencies on  and  reflect that molecular oxygen species are involved in the rate-determining step.

Cation interdiffusion and/or changes in the electronic structure create an electrocatalytically active region (EAR) in the vicinity of the phase boundary between BTM and LSM that exhibits enhanced surface kinetics. Estimates of exchange rates in this 1–40 nm wide area show that the EAR is 2 to 3 orders of magnitude higher than if the entire composite surface was active. Two potential reaction mechanisms for oxygen exchange in BTM–LSM are proposed: (A) the spillover route with step A2 as the *rds* and (B) oxygen exchange within the EAR with step B1 as the *rds*.

Nanostructured BTM–LSM composites with optimized composition have the potential to become state-of-the-art positrode materials for cathodes in solid-state electrochemical cells and membranes for oxygen gas separation. Additionally, the surface and bulk properties of these composites align with the best mixed oxide ion-electron conducting materials for oxygen transport membranes.

## Supporting Information Available

Theoretical and experimental details, reaction pathways, and calculated oxygen partial pressure dependencies for reaction steps in the oxygen exchange mechanism (PDF)
