# Supplementary material for: Superior Oxygen Exchange Kinetics on Bi2O3-Based Mixed Conducting Composites
Source: ACS Phys Chem Au. 2025 Feb 11;5(2):239–48. doi: 10.1021/acsphyschemau.4c00111 (PMC11950862; doi:10.1021/acsphyschemau.4c00111)
Supplement: Supplementary file 1 — pg4c00111_si_001.pdf [file pg4c00111_si_001.pdf]

Supporting Information for

Superior oxygen exchange kinetics on Bi_2O_3-based mixed conducting composites

Linn Katinka Emhjellen[a], Vincent Thoreton[a], Wen Xing[b], Reidar Haugsrud[a]*

[a]Department of Chemistry, Centre for Materials Science and Nanotechnology, University of Oslo, FERMiO, Gaustadalléen 21, NO-0349 Oslo, Norway
[b]SINTEF Industry, Sustainable Energy Technology, Pb. 124 Blindern, NO-0349 Oslo, Norway

*Corresponding authors: reidar.haugsrud@kjemi.uio.no

Table of Contents

S-1. Theory **2**

S-1.1 Pulse Isotope ^{16}O-^{18}O exchange (PIE) 2

S-1.2 Rate expressions 3

S-1.3 Mechanistic interpretations of the p_{O_2} dependency of $\Re_0$ 4

S-2. Experimental 5

S-3. Activation energies for BTM-LSM composites in air 6

S-4. $\Re_{da}$ and $\Re_i$ for BTM and BTM-LSM 50/50 6

S-5. Oxygen exchange rates for BTM 8

S-6. Oxygen exchange coefficient of the EAR 9

S-7. Reaction pathways for the oxygen exchange reaction 12

S-8. Calculation of the oxygen partial pressure dependencies of possible *rds* 13

S-9. Relationship between positrode polarization resistances and $\Re_0$ 15

S-1. Theory [1]

S-1.1 Pulse isotope ^{16}O-^{18}O exchange (PIE)

In PIE [2, 3], an ^{18}O-enriched gas pulse passes through a continuous flowpacked-bed micro-reactor loaded with an oxide powder sample, and the resulting mixture of $^{16}O_2$, $^{16}O^{18}O$ and $^{18}O_2$ are quantified by mass spectrometry (MS) at the outlet. The overall surface exchange rate, $\Re_0$ [mol (O) m^{-2} s^{-1}], is determined by

$$\Re_0 = \frac{\mathrm{F_m}}{\mathrm{S}} \ln\left(\frac{f_{18}^{i}}{f_{18}^{e}}\right) \quad \text{(S-Eq.1)}$$

where f_{18}^{i} and f_{18}^{e} are the ^{18}O isotope fractions in the pulse at the inlet and exit of the reactor, respectively, F_m is the molar flow rate of O_2 through the packed bed, and S the total surface area of the oxide powder. The fraction f_{18} is calculated according to $f_{18} = 0.5f_{34} + f_{36}$.

S-1.2 Rate expressions

Assuming a serial two-step scheme (M1) [2, 4, 5] according to Eq. 3 and Eq.4, the overall surface exchange rate may be expressed as:

$$\Re_0 = \frac{\Re_{da}\Re_{i}}{\Re_{da} + \Re_{i}} = p\Re_{da} \quad \text{(S-Eq.2)}$$

$\Re_{da}$ and $\Re_{i}$ are the rates of oxygen dissociative adsorption and incorporation, respectively. The quantity p varies between 0 and 1 and represents the probability of successful incorporation of both atoms in the O_2 molecule into the oxide bulk.

As evident from S-Eq.2., if $\Re_0$ is heavily dominated by either $\Re_{da}$ or $\Re_{i}$, the faster of these two rates may carry a rather large uncertainty. This is the case for BTM-LSM; $\Re_0$ is heavily dominated by $\Re_{da}$ below 0.05 atm O_2, yielding trustable values of $\Re_{i}$ only at high oxygen pressures (≥ 0.02 atm O_2). In addition, the fitting of the oxygen isotopologue fractions is especially sensitive to the formation of $^{16}O^{18}O$. If the peak corresponding to f_{34} is large (> 0.5) due to isotopic scrambling, i.e., homomolecular exchange, the data is not possible to fit. On the other hand, if the peak is very small due to fast oxygen incorporation, the discrepancy in the

fitting could be substantial. This can be seen in Fig. 1, where the fast incorporation at low oxygen partial pressure leads to small f_{34} peaks and poor fits at 0.002 atm O_2.

The overall surface exchange rate for the parallel two-step scheme (M2) is based on Eq.2 and Eq.4 and may be expressed as

$$\mathfrak{R}_0 = \frac{1}{2}\,\overset{1}{\mathfrak{R}}_{\text{da}}\left(1+\frac{\overset{1}{\mathfrak{R}}_{\text{i}}}{\frac{1}{2}\overset{1}{\mathfrak{R}}_{\text{da}}+\overset{1}{\mathfrak{R}}_{\text{i}}}\right)=\frac{1}{2}\,\overset{1}{\mathfrak{R}}_{\text{da}}(1+p) \qquad \text{(S-Eq.3)}$$

The fraction of oxygen isotopologues in the pulse response for BTM-LSM 50/50 was fitted to both M1 and M2 [2, 4]. As an example, Fig. S-1 displays, $\mathfrak{R}_{0,}$ $\overset{1}{\mathfrak{R}}_{\text{da}}$ and $\overset{1}{\mathfrak{R}}_{\text{i}}$ for BTM-LSM 50/50 as a function of temperature at 0.21 atm and 0.02 atm O_2 using M2 as the fitting model. Both models yielded similar results; incorporation was faster than dissociative adsorption, except above 850 °C in 0.21 atm O_2 for M1 and above 750 °C in 0.21 atm O_2 and 0.05 atm O_2 for M2. Despite significant scatter in the data for $\mathfrak{R}_{\text{i}}$ and $\overset{1}{\mathfrak{R}}_{\text{i}}$ using both models, it is reasonable to conclude that $\mathfrak{R}_{\text{da}}$ and $\overset{1}{\mathfrak{R}}_{\text{da}}$ are more activated than $\mathfrak{R}_{\text{i}}$ and $\overset{1}{\mathfrak{R}}_{\text{i}}$. It should be noted that $\mathfrak{R}_{\text{da}}$ and $\overset{1}{\mathfrak{R}}_{\text{da}}$ do not describe the same process, as $\overset{1}{\mathfrak{R}}_{\text{da}}$ comprises of both dissociative adsorption and incorporation of one oxygen atom. In general, model M1 provided better fits than M2, especially for the f_{34} curve. Therefore, model M1 was chosen for further interpretation of the rates of dissociative adsorption and incorporation. It is worth mentioning that whether M1 or M2 is the most accurate model to describe the oxygen exchange reaction cannot be decided based on PIE data alone [6].

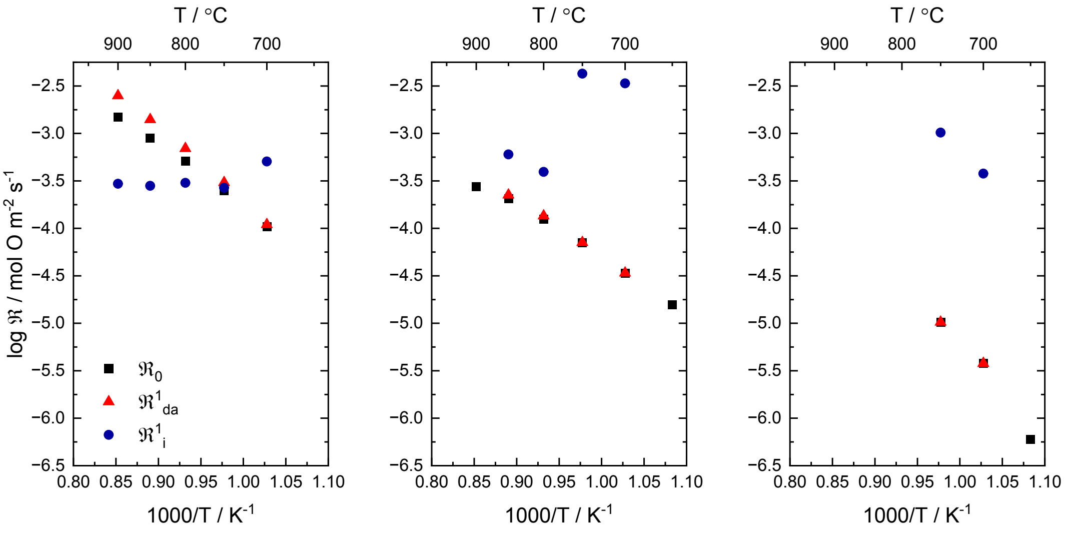

Fig. S-1. $\mathfrak{R}_0$, $\mathfrak{R}_{da}^{1}$ and $\mathfrak{R}_{i}^{1}$ for BTM-LSM 50/50 as a function of temperature in 0.21 (left), 0.02 (middle) and 0.002 (right) atm O_2 using the parallel two-step scheme (M2) as fitting model.

S-1.3 Mechanistic interpretation of the p_{O_2} dependency of $\mathfrak{R}_0$

The surface exchange mechanism can be untangled based on the variation of $\mathfrak{R}_0$ on oxygen partial pressure. Two utmost cases can be considered as rate-limiting: i) dissociative adsorption and ii) oxygen incorporation. The serial two-step scheme (M1) is used here. If dissociative adsorption according to

$$O_2 \rightleftharpoons 2O_{ad}^- + 2h^\bullet \Rightarrow K_{da} = \frac{[O_{ad}^-]^2[h^\bullet]^2}{p_{O_2}} \qquad \text{(S-Eq.4)}$$

determines the oxygen exchange rate, the rate constant may be expressed by the forward reaction rate constant, $\vec{k}$, as

$$\mathfrak{R}_0 = \vec{k}_{da}\, {p_{O_2}}^n \Rightarrow \mathfrak{R}_0 \propto {p_{O_2}}^n \qquad \text{(S-Eq.5)}$$

Since molecular oxygen is involved in the reaction, $n = 1 - m$, where m is related to the influence of point defects on the p_{O_2} exponent, *n*. This requires some defect species to take part in the reaction, with their concentration reducing the slope of the reaction rate with regard to the oxygen partial pressure. In the case where incorporation of a single, adsorbed oxygen atom according to

$$O_{ad}^- + v_O^{\bullet\bullet} \rightleftharpoons O_O^x + h^\bullet \Rightarrow K_i = \frac{[h^\bullet]}{[O_{ad}^-][v_O^{\bullet\bullet}]} \qquad \text{(S-Eq.6)}$$

is the rate determining step (*rds)*, the rate constant may be expressed by the forward reaction rate as

$$\mathfrak{R}_0 = \frac{\vec{k}_i K_{da}^{\frac{1}{2}}[v_O^{\bullet\bullet}]}{[h^\bullet]} {p_{O_2}}^{\frac{1}{2}} \Rightarrow \mathfrak{R}_0 \propto {p_{O_2}}^{0.5-m} \qquad \text{(S-Eq.7)}$$

$\mathfrak{R}_0$ is thus found to be proportional to ${p_{O_2}}^n$ with $n = 0.5 - m$.

This enables a first interpretation of the p_{O_2} dependency of $\mathfrak{R}_0$: Mechanisms containing molecular oxygen of any charge in the *rds* yield $0.5 \leq n \leq 1$, while mechanisms containing only atomic oxygen species yield $n \leq 0.5$ [7, 8].

S-2. Experimental

The surface exchange kinetics of BTM and 60/40, 50/50 and 30/70 vol. % BTM-LSM were investigated by pulse-response isotope exchange (PIE) measurements [2]. Prior to measurements, fine-grained powders of the respective composition were uniaxially pressed (isobaric) to disks of diameter 20 mm. BTM disks were sintered at 950 °C for 10 hrs in air, and BTM-LSM disks were sintered at 1000 °C for 10 hrs in air, yielding relative densities of ~90 %. After sintering, the disks were crushed into coarse powders and sieved to particle sizes of 90-125 μm. All powders were characterized by XRD on a Bruker AXS D8 Discover with Cu $K_{\alpha 1}$ radiation (λ = 1.5406 Å) and Brunauer, Emmett and Teller (BET) surface area measurements on a Monosorb (Quantachrome Instruments).

PIE measurements were performed in the temperature range 30-950 °C in oxygen partial pressures of 0.002 - 0.21. 40-50 mg of powder was loaded into the center of a quartz tube with an inner diameter of 2 mm between two plugs of quartz wool. The length of the packed bed was 3-4 mm. The carrier gas was a $^{16}O_2/N_2$ (21% $^{16}O_2$ in N_2) gas mixture with a flow rate of 50 ml/min (STP), and a six-port valve with a 500 μl sample loop was used for injection of $^{18}O_2$/Ar (21% $^{18}O_2$ in Ar) pulses into the $^{16}O_2/N_2$ gas stream. The pulse response was analyzed at the exit of the reactor using a Pfeiffer QME 220 Mass Spectrometer (MS). The mean residence time of the reactor was 10 ms. Prior to measurements, all powders were pre-annealed at 900 °C for 1 h under flowing carrier gas to remove any adsorbed species, e.g. CO_2 and water, and subsequently cooled to room temperature at a rate of 120 °C/h. The reactor was equilibrated for 40 minutes at each temperature before data collection.

S-3. Activation energies for BTM-LSM composites in air

Table S-1 shows the activation energies of $\Re_0$, $\Re_{da}$ and $\Re_i$ for BTM-LSM composites in air obtained from fitting the data to the serial two-step scheme (M1).

Table S-1. Activation energies of $\Re_0$, $\Re_{da}$ and $\Re_i$ for BTM-LSM composites in air.

Process	BTM-LSM 10-90	BTM-LSM 30-70	BTM-LSM 50-50	BTM-LSM 60-40
$\Re_0$	144	108	138	120
$\Re_{da}$			168	

**$\Re_i$**	72

S-4. $\Re_{da}$ and $\Re_i$ for BTM and BTM-LSM 50/50

Table S-2 shows the apparent activation energies of $\Re_0$, $\Re_{da}$ and $\Re_i$ for BTM-LSM 50/50 at different oxygen partial pressures.

Table S-2. Activation energies of $\Re_0$, $\Re_{da}$ and $\Re_i$ for BTM-LSM 50/50 at different oxygen partial pressures.

Process	Ea at 0.002 atm O_2 (kJ/mol)	Ea at 0.005 atm O_2 (kJ/mol)	Ea at 0.02 atm O_2 (kJ/mol)	Ea at 0.05 atm O_2 (kJ/mol)	Ea at 0.21atm O_2 (kJ/mol)
**$\Re_0$**	149	109	117	113	138
**$\Re_{da}$**	151	111	121	127	168
**$\Re_i$**	89	96	84	75	72

Fig. S-2a) and b) shows $\Re_{da}$ and $\Re_i$ for BTM-LSM 50/50 as a function of oxygen partial pressure, respectively. The p_{O_2} dependence on $\Re_{da}$ follows that of $\Re_0$ within the measured p_{O_2} range. $\Re_i$ on the other hand, is essentially independent of p_{O_2} above 0.02 atm O_2. As mentioned in section 1.3, it follows from theory that the rate constant of reaction steps containing only atomic oxygen species, such as incorporation steps, are proportional to ${p_{O_2}}^n$ with $n \leq 0.5$.

At low oxygen partial pressures (< 0.02 atm O_2), rapid incorporation results in tiny f_{34} peaks that are challenging to fit accurately. Additionally, in this oxygen pressure range, $\Re_0$ is mainly constrained by $\Re_{da}$. Consequently, this introduces significant uncertainty in $\Re_i$, and thus, $\Re i$ values are considered reliable only at higher oxygen pressures (≥0.02 atm O_2; see Supporting Information, section 1.2.). The observed change in slope for $\Re i$ at lower oxygen partial pressures (see Fig. S-2b) is therefore attributed to fitting errors.

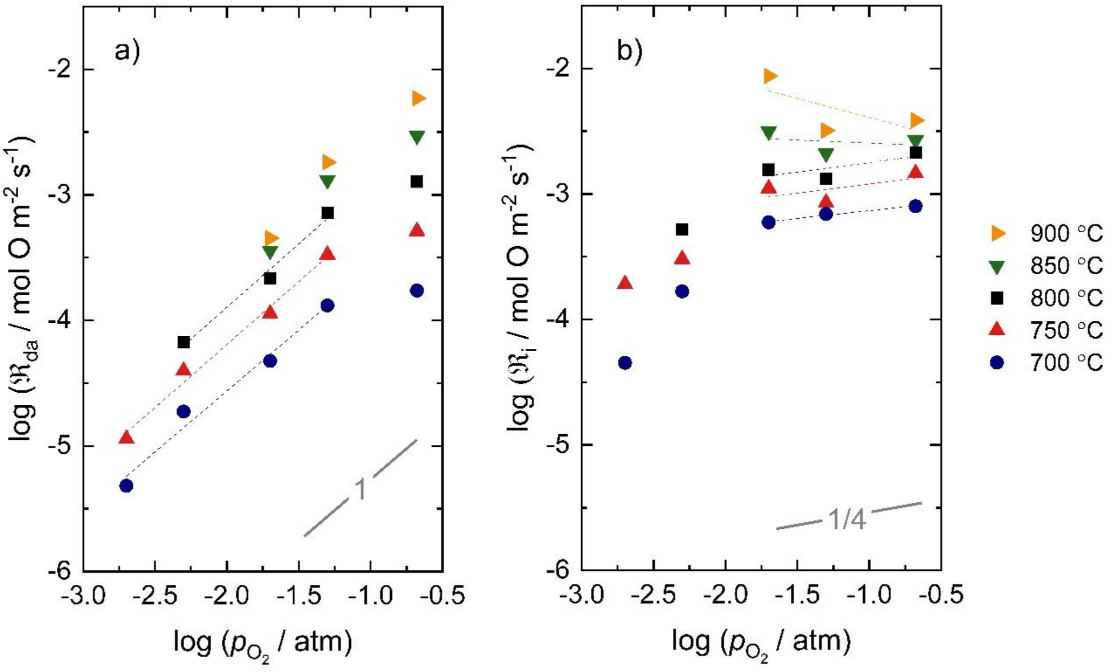

Fig. S-2. p_{O_2} dependency of a) $\Re_{da}$ and b) $\Re_i$ for BTM-LSM 50/50 from 700 to 900°C. Mean slopes with mean standard deviations of 1.00 ± 0.10 for a) and 0.01 ± 0.17 for b), respectively, were obtained from linear regression. Direct proportionality in the log-log representation is indicated as guide for the eye in the lower right corners. The x- and y-axis have the same range in all the graphs.

S-5. Oxygen exchange rates for BTM

Fig. S-3 shows $\Re_0$, $\Re_{da}$, and $\Re_i$ for BTM as a function of temperature at a) 0.02 and b) 0.21 atm O_2.

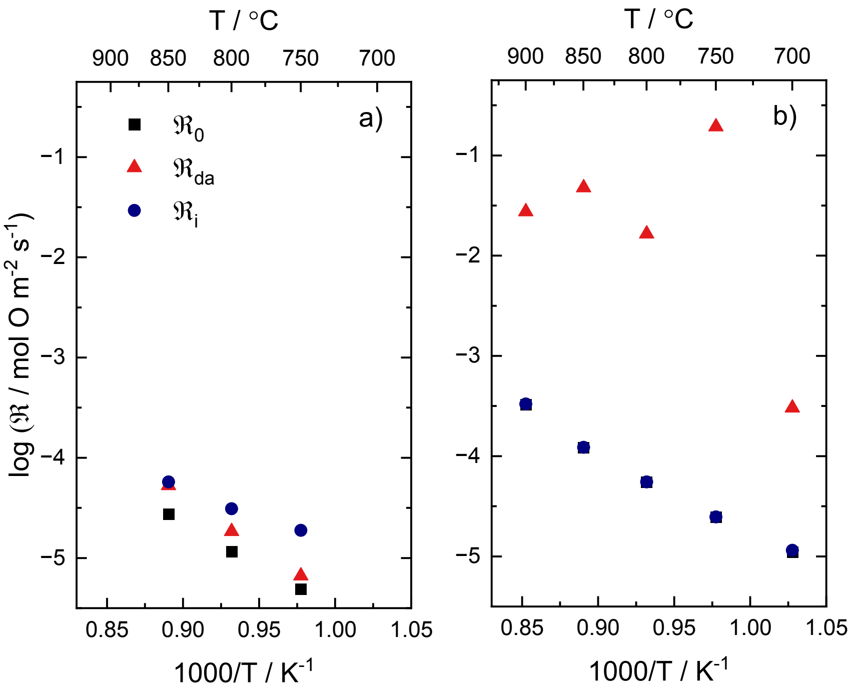

Fig. S-3. $\Re_0$, $\Re_{da}$, and $\Re_i$ for BTM at a) 0.02 and b) 0.21 atm O_2. The data was fitted with the serial two-step scheme (M1).

Fig. S-4 shows $\Re_{da}$, and $\Re_i$ for BTM and BTM-LSM 50/50 as a function of oxygen partial pressure at different temperatures. Both $\Re_{da}$, and $\Re_i$ are enhanced in the composite as compared to single-phase BTM.

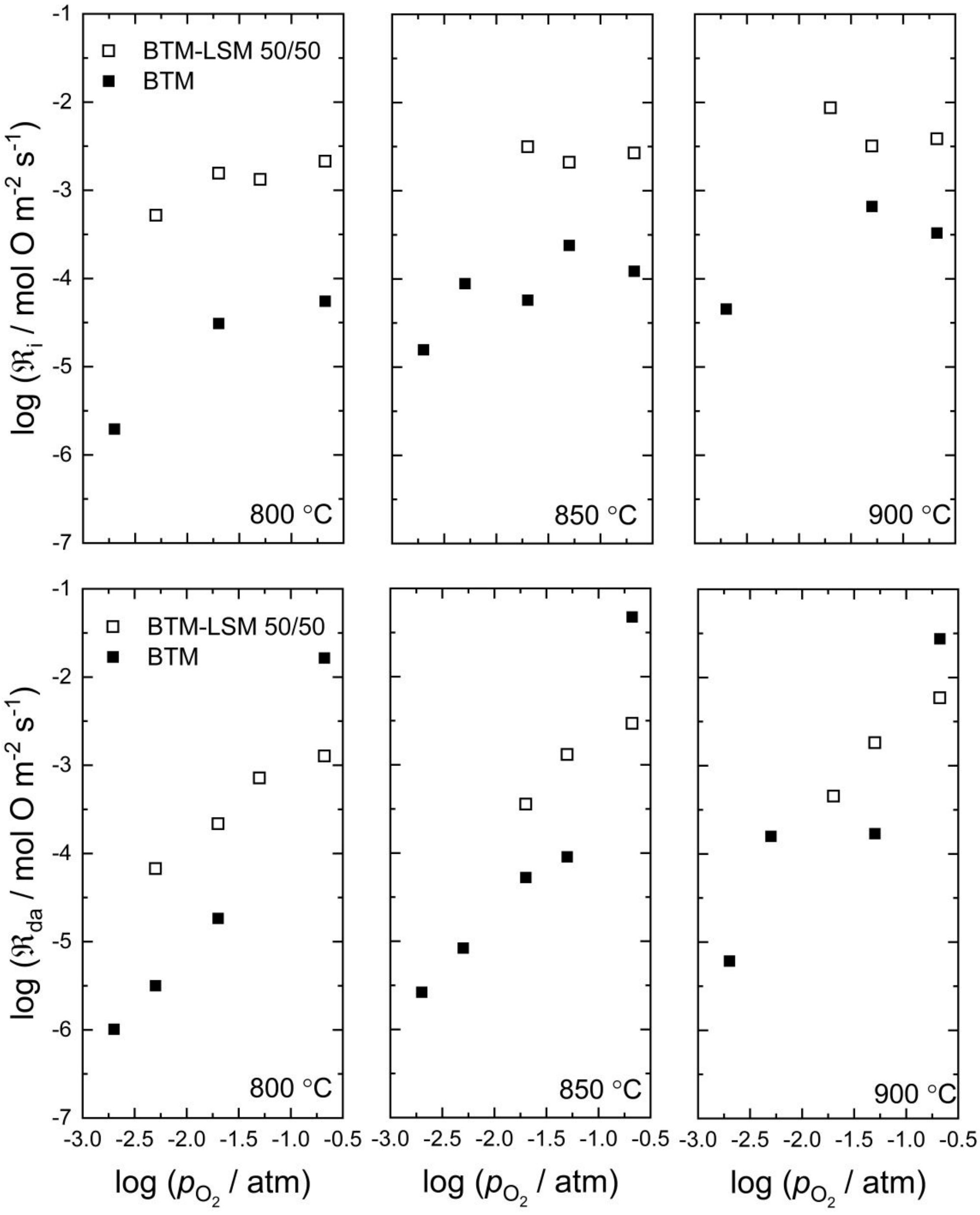

Fig. S-4. Rates of dissociative adsorption, $\Re_{da}$, and incorporation, $\Re_i$, for BTM and BTM-LSM 50/50 at 800, 850 and 900 °C.

S-6. Oxygen exchange coefficient of the EAR

The oxygen exchange coefficient for BTM-LSM can be represented as a sum of the oxygen exchange coefficients k of the individual phases and the electrocatalytically active region (EAR) with respect to their relative surface areas [9]:

$$k_{BTM-LSM} = S_{BTM}k_{BTM} + S_{LSM}k_{LSM} + S_{EAR}k_{EAR} \quad \text{(S-Eq.8)}$$

where S is the ratio between the area of the respective phase and the total surface area of the composite. Further,

$$S_{EAR} = \frac{xL_{PB}}{S_{TOT}} \quad \text{(S-Eq.9)}$$

where L_{PB} is the length of the phase boundary between BTM and LSM at the composite surface. L_{PB} was estimated from SEM micrographs of the composite surface by means of the graphic pixel-based Canny edge detecting algorithm in Python [10]. Then the number of phase boundary pixels were translated into the relative area of the EAR, S_{EAR}, to the total surface area, S_{TOT}, with an assumed width, x, of the EAR. Fig. S-4 and S-5 shows a SEM image of the surface of BTM-LSM 50-50 and 20 randomly selected surface areas with the detected phase boundaries.

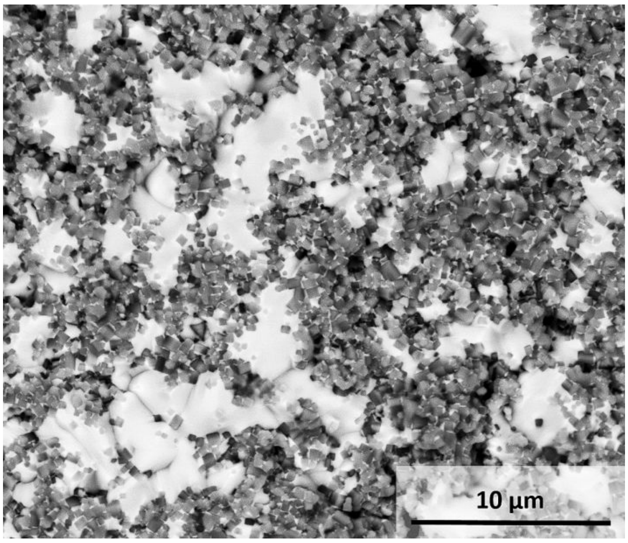

Fig. S-4. SEM image of the surface of BTM-LSM 50/50. The light grey grains are BTM and the dark grey grains are LSM.

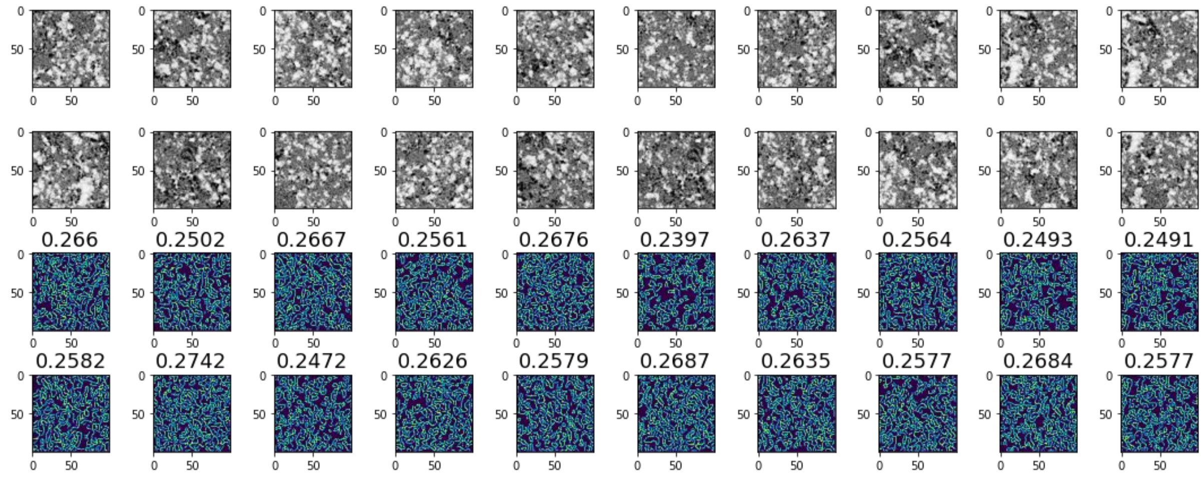

Fig. S-5. 20 randomly selected areas with size 100 x 100 pixels from the SEM image of the surface of BTM-LSM 50/50 (top two rows) and the detected phase boundaries using the Canny edge detection algorithm in Python (bottom two rows). The calculated edge pixel to the total pixel ratio is shown on top of every phase boundary image.

Despite the variations in BTM/LSM volume ratios, all investigated BTM-LSM compositions exhibited similar TPB areas, i.e., a consistent TPB to surface area ratio of approximately 0.25 [11]. Given the minor differences in k and TPB area, it is challenging to assess the impact of TPB area on the oxygen exchange rate of BTM-LSM.

In the composite, the BTM grains are significantly larger than the LSM grains, likely due to partial surface melting that accelerates agglomeration. In ref. [11], to increase the TPB area by reducing the grain size of BTM, the powders were milled using a planetary ball mill for 1 hour before sintering. Although milling reduced the BTM grain size by a factor of approximately 5, the oxygen exchange rate in the BTM-LSM 50/50 composite with smaller BTM grains showed only a minimal improvement.

Fig. S-6 shows the oxygen exchange coefficient of BTM-LSM 50/50 measured by PIE compared to EARs of 1 nm and 40 nm widths. Data for nanostructured LSM with 1 nm wide grain boundaries taken from ref. [12] and the fundamental upper limit of k as determined by the flux of oxygen molecules in the gas phase taken from ref. [13] are also included.

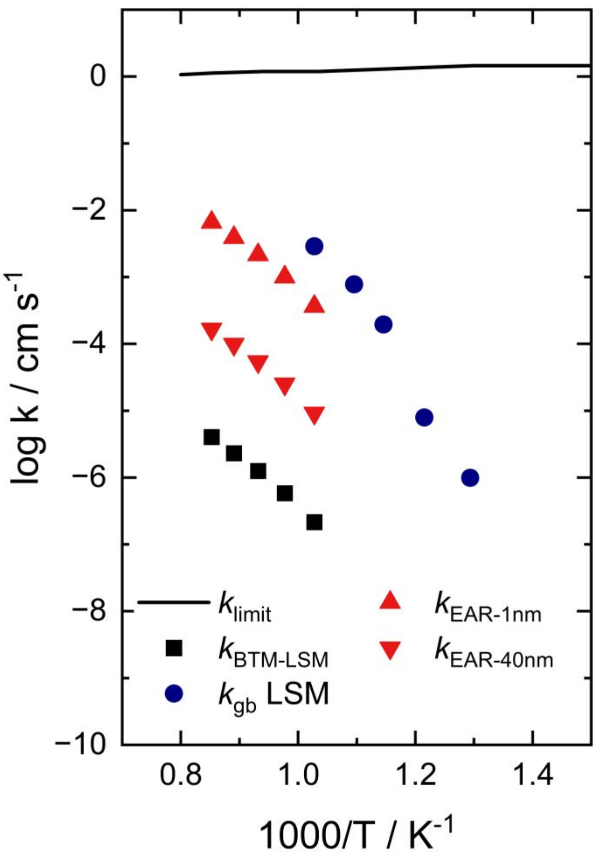

Fig. S-6. Calculated oxygen exchange coefficient, *k*, for BTM-LSM 50/50 with 1 nm wide and 40 nm EAR compared to nanostructured pulse layer deposition (PLD) thin-film LSM with 1 nm wide grain boundaries [12] and a fundamental upper-limit of *k* set by the flux of oxygen molecules in the gas phase at ~0.21 atm O_2 [13].

S-7. Reaction pathways for the oxygen exchange reaction

Two possible reaction pathways for the oxygen exchange were considered for BTM-LSM depending on which phase dominates the dissociative adsorption of oxygen; A) the spillover route - dissociative adsorption on LSM followed by surface diffusion of $O^{-}$to BTM where incorporation occurs and B) dissociative adsorption and incorporation in the electrocatalytically active region (EAR).

Reaction pathway A: The spillover route - dissociative adsorption on LSM and incorporation in BTM

Reaction pathway A comprises of dissociative adsorption on LSM and surface diffusion of O^{-} to BTM where incorporation occurs ("surface path"). A combination of experimental and computational studies have shown that the dissociative adsorption on the surface of LSM most

likely occurs via O_2 adsorption followed by dissociation of $O_{2,ad}$, $O^-_{2,ad}$ or $O^{2-}_{2,ad}$ to O^-_{ad}, without the assistance of oxygen vacancies [14]. Further, the dissociation energies of $O^-_{2,ad}$ and $O^{2-}_{2,ad}$ are significantly lower than that of $O_{2,ad}$, hence some charge transfer steps occurs before dissociation [15]. On these bases, the exchange reaction for pathway A is a serial mechanism (M1), and may be divided into the following successive reaction steps with the corresponding equilibrium constants;

**Step A1**	$O_2 \rightleftharpoons O_2^- + h^\bullet$	$\Rightarrow K_1 = \frac{[O_2^-][h^\bullet]}{p_{O_2}}$	(S-Eq.10)
**Step A2**	$O_2^- \rightleftharpoons 2O^- + h^\bullet$	$\Rightarrow K_2 = \frac{[O^-]^2[h^\bullet]}{[O_2^-]}$	(S-Eq.11)
**Step A3**	$O^-_{LSM} \rightleftharpoons O^-_{BTM}$	$\Rightarrow K_3 = \frac{[O^-_{BTM}]}{[O^-_{LSM}]}$	(S-Eq.12)
**Step A4**	$O^- + v_O^{\bullet\bullet} \rightleftharpoons O_O^x + h^\bullet$	$\Rightarrow K_4 = \frac{[h^\bullet]}{[O^-][v_O^{\bullet\bullet}]}$	(S-Eq.13)

Steps A1 and A2 are part of the dissociative adsorption reaction, while step A3 reflects surface diffusion of adsorbed oxygen species on LSM to BTM. Step A4 describes the incorporation of oxygen into BTM.

Since fitting the data to the serial two-step scheme (M1) revealed that incorporation is faster than dissociative adsorption ($\Re_i \gg \Re_{da}$) and the overall p_{O_2} dependency of $\Re_0$ indicates that molecular oxygen species are involved in the *rds*, step A1 and A2 are the most probable alternatives for being limiting steps in pathway A.

As mentioned, the "bulk path" may become relevant in certain conditions. This would imply that step A3 vanishes and that the incorporation in step A4 occurs in LSM as opposed to BTM. However, the rate-limiting steps (step A1 and A2, dissociative adsorption) are the same for both paths.

Reaction pathway B: Dissociative adsorption and incorporation on the BTM side of the EAR

Here, the oxygen exchange reaction occurs via the parallel two-step scheme, M2 (see section 1.2). This implies that one oxygen atom is immediately incorporated upon adsorption. The other will either incorporate or recombine with a lattice oxygen. M2 may be divided into the following successive reaction steps with the corresponding equilibrium constants;

**Step B1**	$O_2 \rightleftharpoons O_2^- + h^\bullet$	$\Rightarrow K_1 = \frac{[O_2^-][h^\bullet]}{p_{O_2}}$	(S-Eq.14)
**Step B2**	$O_2^- + v_O^{\bullet\bullet} \rightleftharpoons O^- + O_O^x + 2h^\bullet$	$\Rightarrow K_2 = \frac{[O^-][h^\bullet]^2}{[O_2^-][v_O^{\bullet\bullet}]}$	(S-Eq.15)
**Step B3**	$O^- + v_O^{\bullet\bullet} \rightleftharpoons O_O^x + h^\bullet$	$\Rightarrow K_{3a} = \frac{[h^\bullet]}{[O^-][v_O^{\bullet\bullet}]}$	(S-Eq.16)

Since the overall p_{O_2} dependency of $\Re_0$ indicate that molecular oxygen species are involved in the *rds*, the pure incorporation step B3 cannot be limiting. This was supported by fitting the data to the parallel two-step scheme (M2), revealing that incorporation is faster than dissociative adsorption ($\overset{1}{\Re_i} > \overset{1}{\Re_{da}}$), except above 750 °C in 0.21 atm O_2 and 0.05 atm O_2.

S-8. Calculation of the oxygen partial pressure dependencies of possible *rds* [7]

The p_{O_2} dependency of $\Re_0$ may be influenced by defect concentrations. The bulk concentration of oxygen vacancies in BTM is essentially p_{O_2} independent under the experimental conditions. The concentration of electronic species vary according to $[e^-]^{-1} \sim [h^\bullet] \propto p_{O_2}{}^{\frac{1}{4}}$ for BTM and $[e^-]^{-1} \sim [h^\bullet] \propto p_{O_2}{}^0$ for LSM [16, 17]. The p_{O_2} dependency of the defects in the proposed reaction pathways may differ depending on which phase the dominating defects originate from.

Pathway A is dominated by oxygen vacancies in BTM and electron holes in LSM ($[v_O^{\bullet\bullet}] \propto p_{O_2}{}^0$, $[e^-]^{-1} \sim [h^\bullet] \propto p_{O_2}{}^0$, respectively) [16, 17]. The reaction rates calculated for each step being the *rds* are listed in table S-3. The derivation of the oxygen partial pressure dependency of $\Re_0$ is carried out in the forward direction.

Table S-3. Possible steps and reaction rates in reaction pathway A. Reaction rates $\Re$ are calculated for each step being the *rds*. K_f denote the equilibrium constant of all preceding reaction steps.

**Step A1**	$\boldsymbol{\Re = \vec{k} K_f p_{O_2}}$	$\boldsymbol{\Re_0 \propto p_{O_2}{}^1}$
**Step A2**	$\Re = \vec{k} K_f \frac{1}{[h^\bullet]} p_{O_2}$	$\Re_0 \propto p_{O_2}{}^1$
**Step A3**	$\Re = \vec{k} K_f \frac{1}{[h^\bullet]} p_{O_2}{}^{1/2}$	$\Re_0 \propto p_{O_2}{}^{1/2}$
**Step A4**	$\Re = \vec{k} K_f \frac{[v_O^{\bullet\bullet}]}{[h^\bullet]} p_{O_2}{}^{1/2}$	$\Re_0 \propto p_{O_2}{}^{1/2}$

For pathway B, we presume that the defect chemistry of BTM dominates ($[\mathrm{v_O^{\bullet\bullet}}] \propto p_{\mathrm{O_2}}{}^{0}$, $[\mathrm{e^-}]^{-1} \sim [\mathrm{h^\bullet}] \propto p_{\mathrm{O_2}}{}^{\frac{1}{4}}$) [16]. The reaction rates calculated for each step being the *rds* are listed in table S-4.

Table S-4. Possible steps and reaction rates in reaction pathway B. Reaction rates $\mathfrak{R}$ are calculated for each step being the *rds*. $\mathrm{K_f}$ denote the equilibrium constant of all preceding reaction steps.

**Step B1**	$\mathfrak{R} = \vec{k}K_{\mathrm{f}}p_{\mathrm{O_2}}$	$\mathfrak{R}_0 \propto p_{\mathrm{O_2}}{}^{1}$
**Step B2**	$\mathfrak{R} = \vec{k}K_{\mathrm{f}}\frac{[\mathrm{v_O^{\bullet\bullet}}]}{[\mathrm{h^\bullet}]}p_{\mathrm{O_2}}$	$\mathfrak{R}_0 \propto p_{\mathrm{O_2}}{}^{3/4}$
**Step B3**	$\mathfrak{R} = \vec{k}K_{\mathrm{f}}\frac{[\mathrm{v_O^{\bullet\bullet}}]^2}{[\mathrm{h^\bullet}]^3}p_{\mathrm{O_2}}$	$\mathfrak{R}_0 \propto p_{\mathrm{O_2}}{}^{1/4}$

It should be noted that the transfer of negative charges to oxygen species takes place by consumption of an electron from the conduction band or the valence band (formation of holes). Since electrons and holes are interrelated by intrinsic ionization equilibrium (n=p), and as long as the charge transfer occurs in the preceding or succeeding steps rather than in the *rds*, the type cannot be distinguished.

The charge transfer steps include electrons or electron holes (valence band electrons) depending on the defect structure of the specific oxide. Since both BTM and LSM are *p*-type conductors, the charge transfer reactions would most likely occur via electrons from the valence band.

S-9. Relationship between positrode polarization resistances and $\mathfrak{R}_0$

The polarization resistance R_p from an EIS sweep measured at OCV may be directly related to $\mathfrak{R}_0$ via

$$\mathfrak{R}_0 = \frac{\mathrm{R}T}{4F^2R_p} \qquad \text{(S-Eq.17)}$$

where R, T and F are the gas constant, temperature and Faraday's constant, respectively [18, 19]. It should be noted that although electrode polarization resistances from EIS and oxygen exchange rates from isotope measurements are conceptually comparable, it is not straightforward in reality, as different material combinations (e.g., the presence of an

electrolyte when measuring electrodes in a symmetrical cell) and measurement conditions may alter the rate-determining step [20].

References

1. Bouwmeester, H.J.M. and A.J. Burggraaf, *Chapter 10 Dense ceramic membranes for oxygen separation*, in *Membrane Science and Technology*, A.J. Burggraaf and L. Cot, Editors. 1996, Elsevier. p. 435-528.
2. Bouwmeester, H.J.M., et al., *A novel pulse isotopic exchange technique for rapid determination of the oxygen surface exchange rate of oxide ion conductors.* Physical Chemistry Chemical Physics, 2009. **11**(42): p. 9640-9643.
3. Yoo, C.-Y., B.A. Boukamp, and H.J.M. Bouwmeester, *Oxygen surface exchange kinetics of erbia-stabilized bismuth oxide.* Journal of Solid State Electrochemistry, 2011. **15**(2): p. 231-236.
4. den Otter, M.W., B.A. Boukamp, and H.J.M. Bouwmeester, *Theory of oxygen isotope exchange.* Solid State Ionics, 2001. **139**(1): p. 89-94.
5. Boukamp, B.A., et al. *Surface oxygen exchange kinetics in oxide-ion conducting solids*. in *Materials Research Society Symposium Proceedings*. 1993.
6. Yoo, C.-Y. and H.J.M. Bouwmeester, *Oxygen surface exchange kinetics of $SrTi_{1-x}Fe_xO_{3-\delta}$ mixed conducting oxides.* Physical Chemistry Chemical Physics, 2012. **14**(33): p. 11759-11765.
7. Merkle, R. and J. Maier, *Oxygen incorporation into Fe-doped $SrTiO_3$: Mechanistic interpretation of the surface reaction.* Physical Chemistry Chemical Physics, 2002. **4**(17): p. 4140-4148.
8. Schaube, M., R. Merkle, and J. Maier, *Oxygen exchange kinetics on systematically doped ceria: a pulsed isotope exchange study.* Journal of Materials Chemistry A, 2019. **7**(38): p. 21854-21866.
9. Farlenkov, A.S., et al., *Particle coarsening influence on oxygen reduction in LSM–YSZ composite materials.* Fuel Cells, 2015. **15**(1): p. 131-139.
10. Canny, J., *A computational approach to edge detection.* IEEE Transactions on Pattern Analysis and Machine Intelligence, 1986. **6**: p. 679-698.
11. Emhjellen, L.K., et al., *Oxygen permeability and surface kinetics of composite oxygen transport membranes based on stabilized δ-Bi_2O_3.* Journal of Membrane Science, 2022. **660**: p. 120875.
12. Saranya, A.M., et al., *Engineering mixed ionic electronic conduction in $La0.8Sr_{0.2}MnO_{3+\delta}$ nanostructures through fast grain boundary oxygen diffusivity.* Advanced Energy Materials, 2015. **5**(11): p. 1500377.
13. De Souza, R.A., *Limits to the rate of oxygen transport in mixed-conducting oxides.* Journal of Materials Chemistry A, 2017. **5**(38): p. 20334-20350.
14. Wang, L., et al., *Oxygen exchange kinetics on solid oxide fuel cell cathode materials—general trends and their mechanistic interpretation.* Journal of Materials Research, 2012. **27**(15): p. 2000-2008.
15. De Souza, R.A., *A universal empirical expression for the isotope surface exchange coefficients (k*) of acceptor-doped perovskite and fluorite oxides.* Physical Chemistry Chemical Physics, 2006. **8**(7): p. 890-897.
16. Bouwmeester, H.J.M., et al., *Oxygen semipermeability of erbia-stabilized bismuth oxide.* Solid State Ionics, 1992. **53-56**: p. 460-468.
17. Mizusaki, J., et al., *Electronic conductivity, Seebeck coefficient, defect and electronic structure of nonstoichiometric $La_{1-x}Sr_xMnO_3$.* Solid State Ionics, 2000. **132**(3): p. 167-180.
18. Fleig, J., et al., *Oxygen Reduction Kinetics of Lanthanum Manganite (LSM) Model Cathodes: Partial Pressure Dependence and Rate-Limiting Steps.* Fuel Cells, 2008. **8**(5): p. 330-337.
19. Fleig, J. and J. Maier, *The polarization of mixed conducting SOFC cathodes: Effects of surface reaction coefficient, ionic conductivity and geometry.* Journal of the European Ceramic Society, 2004. **24**(6): p. 1343-1347.

20. Maier, J., *On the correlation of macroscopic and microscopic rate constants in solid state chemistry.* Solid State Ionics, 1998. **112**(3): p. 197-228.
